# Culturally Adapted Hypertension Education (CAHE) to Improve Blood Pressure Control and Treatment Adherence in Patients of African Origin with Uncontrolled Hypertension: Cluster-Randomized Trial

**DOI:** 10.1371/journal.pone.0090103

**Published:** 2014-03-05

**Authors:** Erik J. A. J. Beune, Eric P. Moll van Charante, Leo Beem, Jacob Mohrs, Charles O. Agyemang, Gbenga Ogedegbe, Joke A. Haafkens

**Affiliations:** 1 Department of General Practice, Division of Clinical Methods and Public Health, Academic Medical Center, University of Amsterdam, Amsterdam, The Netherlands; 2 Department of Public Health, Division of Clinical Methods and Public Health, Academic Medical Center, University of Amsterdam, Amsterdam, The Netherlands; 3 Center for Healthful Behavior Change, Division of General Internal Medicine, Department of Medicine, New York University School of Medicine, New York, New York, United States of America; Catholic University of Sacred Heart of Rome, Italy

## Abstract

**Objectives:**

To evaluate the effect of a practice-based, culturally appropriate patient education intervention on blood pressure (BP) and treatment adherence among patients of African origin with uncontrolled hypertension.

**Methods:**

Cluster randomised trial involving four Dutch primary care centres and 146 patients (intervention n = 75, control n = 71), who met the following inclusion criteria: self-identified Surinamese or Ghanaian; ≥20 years; treated for hypertension; SBP≥140 mmHg. All patients received usual hypertension care. The intervention-group was also offered three nurse-led, culturally appropriate hypertension education sessions. BP was assessed with Omron 705-IT and treatment adherence with lifestyle- and medication adherence scales.

**Results:**

139 patients (95%) completed the study (intervention n = 71, control n = 68). Baseline characteristics were largely similar for both groups. At six months, we observed a SBP reduction of ≥10 mmHg -primary outcome- in 48% of the intervention group and 43% of the control group. When adjusted for pre-specified covariates age, sex, hypertension duration, education, baseline measurement and clustering effect, the between-group difference was not significant (OR; 0.42; 95% CI: 0.11 to 1.54; P = 0.19). At six months, the mean SBP/DBD had dropped by 10/5.7 (SD 14.3/9.2)mmHg in the intervention group and by 6.3/1.7 (SD 13.4/8.6)mmHg in the control group. After adjustment, between-group differences in SBP and DBP reduction were −1.69 mmHg (95% CI: −6.01 to 2.62, P = 0.44) and −3.01 mmHg (−5.73 to −0.30, P = 0.03) in favour of the intervention group. Mean scores for adherence to lifestyle recommendations increased in the intervention group, but decreased in the control group. Mean medication adherence scores improved slightly in both groups. After adjustment, the between-group difference for adherence to lifestyle recommendations was 0.34 (0.12 to 0.55; P = 0.003). For medication adherence it was −0.09 (−0.65 to 0.46; P = 0.74).

**Conclusion:**

This intervention led to significant improvements in DBP and adherence to lifestyle recommendations, supporting the need for culturally appropriate hypertension care.

**Trial Registration:**

Controlled-Trials.com ISRCTN35675524

## Introduction

In Western countries, people of African descent have a higher prevalence of hypertension (HTN) and HTN-related cardiovascular morbidity and mortality than people of European origin (henceforth, white) [1]–[3]. The Netherlands has two major populations of African descent: African-Surinamese, who immigrated to the Netherlands after the former Dutch colony of Suriname gained its independence in 1975 (hereafter referred to as Surinamese), and Ghanaians, who immigrated to the Netherlands in the 1970s and 1980s during the economic downturn in West Africa. Dutch studies reported prevalence rates of HTN of 47% among Surinamese, 55% among Ghanaians compared with 38% among whites [4], [5]. While these studies found no differences in HTN awareness and treatment rates among the three ethnic groups, among treated hypertensives, blood pressure (BP) control rates varied from respectively 37% for the Surinamese, 33% for the Ghanaians and 47% for the whites [5], [6]. This demonstrates that there is a need to address barriers to BP control among Surinamese and Ghanaians who are treated for HTN in the Netherlands.

Poor adherence to prescribed medication and lifestyle recommendations has been identified as the most important modifiable cause for disparities in BP control and, consequently, the occurrence of HTN-related complications [7]. Enhancing patient adherence to therapeutic measures is an essential first step towards reducing the observed ethnic disparities in BP control [3], [7]–[10].

In the Netherlands, general practitioners (GPs) play an important role in the treatment of HTN. Both national and international primary care guidelines recommend patient education as a means of enhancing patients' motivation and ability to adhere to HTN treatment goals [11], [12]. Patient perceptions about the onset, symptoms, pathophysiology, course and treatment of HTN can differ substantially from those of their healthcare providers [13], and this may have a profound impact on adherence to treatment [14]–[16]. HTN care providers are therefore advised to employ “patient-centred” educational approaches that allow them to explore the individual beliefs and needs of their patients, and to find common ground regarding treatment [17]. The Dutch guidelines recommend motivational interviewing according to the “5 A's” framework (i.e. ask, assess, advise, assist and arrange) as the preferred method for HTN counselling for all patients [18]. Studies from the UK and the US have shown that patient beliefs about HTN and treatment can differ between ethnic groups [19]–[23]. We have also found this in our previous studies, which focused on white Dutch, Surinamese and Ghanaian hypertensive patients living in the Netherlands [24]–[26]. In the patient education literature there is increasing theoretical support for the notion that culturally adapted educational interventions may be better suited to support ethnic minority patients in chronic disease management than generic interventions [27]–[29]. Culturally appropriate patient education typically combines the principle of “patient-centred” care with that of “culturally competent” care [30]. To date, the effect of practice-based, culturally adapted patient education on BP control and treatment adherence remains, however, largely untested among hypertensive blacks living in Western countries [27], [28], [31], [32].

In previous studies we developed a protocol to facilitate the delivery of culturally adapted hypertension education (CAHE) and we identified provider-based barriers and enablers influencing the implementation of CAHE by Dutch primary care practices [24]–[26], [33]. In this paper we report the results of a cluster randomised controlled trial that evaluated the effect of CAHE compared to usual care, on BP control and adherence to lifestyle recommendations and medications. The trial was carried out in Dutch primary health care centres (PHCC) among hypertensive Surinamese and Ghanaian patients with poor BP control.

## Methods

The protocol for this trial and supporting CONSORT checklist are available as supporting information; see Checklist S1 and Protocol S1

### Study design

The study methods have been described elsewhere [34]. We conducted a cluster randomised trial in Dutch PHCCs, comparing CAHE (intervention) to usual care (control) with the health centre as the unit of randomisation. The intervention was delivered to patients individually and the outcome measures were also assessed individually. Cluster randomisation was chosen to prevent contamination between the two conditions within the health care centres. Patients in the control sites received standard hypertension care and education as recommended by the Dutch clinical guidelines for GPs [35]. In addition to standard care, patients in the intervention sites received three CAHE sessions delivered by a trained practice nurse (PN), during the second, eighth and twentieth week post-baseline. Data were collected at baseline and at six months follow-up.

### Setting and patients

Four PHCCs in Southeast Amsterdam took part in the study. The percentage of Surinamese and Ghanaian residents in this area is relatively high (33% and 9% respectively). PHCCs were eligible to participate if they provided hypertension care according to the Dutch clinical guidelines [35], maintained an electronic medical record (EMR) for their patients and were not participating in other studies to improve cardiovascular risk management.

Potentially eligible patients were identified via anonymised EMRs from the selected PHCCs. These patients had to meet the following eligibility criteria: aged 20 or older, having a diagnosis of HTN based on the International Classification of Primary care codes K85, K86 or K87, and having SBP≥140 mmHg at the last office visit. Patients were excluded if they had type 1 or type 2 diabetes, since diabetes requires additional care [35]. Because Dutch EMRs do not provide information on ethnicity, GPs were asked to identify Ghanaian and Surinamese patients on their list of potentially eligible patients. They were also asked to exclude patients whom they judged unfit to participate in the study. Next, the PHCCs were randomly assigned to either the intervention (n = 2) or the control status (n = 2) by a member of the Academic Medical Center's Data Management Services team who was blinded to the study. Eligible patients were sent a letter with information about the study and an invitation to participate, co-signed by their GP. Study participants were promised an incentive payment of 40 Euros upon completion of the final assessments. If patients failed to respond, a reminder was sent after two weeks. Patients who expressed interest in participating were contacted by telephone by a research assistant (RA) to verify the ethnic background by self-identification [36], and their ability to speak and understand basic English or Dutch. The RA made an appointment with those who fulfilled all inclusion criteria to obtain informed consent and to conduct baseline measurements.

### Ethics

In accordance with the Declaration of Helsinki, written informed consent was obtained from all participants. The study protocols were approved by the medical ethics committee of the Academic Medical Center in Amsterdam (protocol ID MEC 09/070 # 09.17.0725) and CCMO (NL27507.018.09). This trial is registered at the ISRCTN Register under registration number ISRCTN35675524 (http://www.controlled-trials.com/ISRCTN35675524).

### Intervention

Details of the intervention are described elsewhere [33], [34]. Briefly, patients at the intervention sites received usual care plus (i) three structured 30-minute culturally appropriate counselling sessions at 2 weeks, 8 weeks and at 20 weeks after baseline assessment; (ii) culturally appropriate written educational materials; and (iii) if applicable, referrals to neighbourhood facilities, such as walking clubs and health food stores, that support patients in adopting healthier lifestyles and are suitable for Surinamese and Ghanaian people. A trained PN conducted all sessions. We assigned the task of patient educator to a PN because in the Dutch primary care system, hypertension education is generally delivered by PNs working under the supervision of GPs. Due to the behavioural nature of the intervention, neither the patients nor the PN were blinded to the intervention. Table 1 and 2 provide an overview of the CAHE protocol.

**Table 1:** Summary of three culturally adapted hypertension education sessions.

SESSIONS	CONTENT SESSIONS			
Main topic	Method and topics to be addressed			
	Elicit patient perspective, using culturally-sensitive framework	Inform patient about medical perspective	Reach consensus about:	Establish:
***SESSION 1***				
**Establishing communication, identifying barriers and rapport**	Experience of (culturally specific) communication barriers (i)			
**What is hypertension?**	What is hypertension? (i)	Discuss hypertension (ii)	What is hypertension? (iii)	Potential barriers/facilitators for achieving treatment goals (i, iii)
	Hypertension treatment and goals? (i)	Discuss treatment goals (ii)	Patient's treatment goals? (iii)	Goal for next 3 months
***SESSION 2 AND 3***				
**How to achieve hypertension treatment goals?**	Experience of (culturally specific) barriers/enablers in achieving patient's hypertension treatment goals: medication use and lifestyle changes (i)	Discuss patient's current: (i, ii, iii) BP measurement, Self-reported medication and lifestyle adherence	What feasible steps are needed to maintain/achieve treatment goals? (iii)	Potential barriers/facilitators for achieving treatment goals (i, iii)
		Discuss patient's treatment goals		Goal for next 3 months

(i) Using culturally-sensitive framework for eliciting a patient's explanatory model of hypertension (table 2); (ii) using information from hypertension guidelines [35]. (iii) using “5 As” method [18].

**Table 2:** Culturally-sensitive framework for eliciting a patient's explanatory model of hypertension^a^.

**COMMUNICATION**
Determine how a patient wants to be addressed (formally or informally)
Determine the patient's preferred language for speaking and reading (Dutch or another language)
Use this information in your interaction with the patient
**INTRODUCTION**
It is often difficult for us (care providers) to give advice about hypertension and how to manage it if we are unfamiliar with our patients' views and experiences. For this reason I would like to ask you some questions to learn more about your own views on hypertension and its treatment.
**ELICIT PERONAL VIEWS ON HYPERTENSION AND ITS TREATMENT**
**a) Understanding**
What do you understand hypertension to mean?
**b) Causes**
What do you think has caused your hypertension? Why has it occurred now/when it did; why to you?
**c) Meaning and symptoms**
What does it mean to you to have hypertension?
Do you notice anything about your hypertension? How do you react in this case?
**d) Duration and consequences**
How do you think your hypertension will develop further? How severe is it?
What consequences do you think your hypertension may have for you (physical, psychological, social)?
**e) Treatment**
What types of treatment do you think would be useful?
What does the prescribed therapeutic measurement(s) mean to you?
**ELICIT CONTEXTUAL INFLUENCES ON HYPERTENSION MANAGEMENT**
**a) Social**
Do you speak with family/community members about your hypertension? How do they react?
Do family/community members help you or make it difficult for you to manage hypertension? Please explain.
**b) Culture/Religion**
Are there any cultural issues/religious issues that may help you or make it difficult for you to manage hypertension? Please explain.
**c) Migration**
Are any issues related to your position as an immigrant making it difficult for you to manage hypertension? Please explain.
**d) Finance**
Are any issues related to your financial situation making it difficult for you to manage hypertension? Please explain

a Based on Kleinman's Explanatory Model format [13], and on our previous studies [24]–[26].

### Outcomes and measurements

The primary outcome was the between-group difference in the proportion of patients with a SBP reduction of at least 10 mmHg at 6 months. The secondary outcomes were the mean between-group differences in changes in SBP and DBP and adherence to lifestyle and medication recommendations from baseline to 6 months.

BP was measured three times using an automated BP monitor (Omron 705-IT), after the patient had been seated for 5 minutes. The average of the last two readings was used to calculate the SBP and DBP. Adherence with respect to lifestyle recommendations was assessed with a scale that was based on the four-item Morisky scale [37]. This scale contains three questions: 1) Have you been advised by your PN/GP about smoking, nutrition, alcohol, weight control and/or physical activity (Yes/No)? 2) If yes, what advice was given? 3) To what extent did you follow this advice (range: never (1) – always (4))? On the basis of answers to these questions a composite score for adherence to lifestyle recommendations was computed (range: 1–4), with 4 indicating full adherence and lower scores indicating lower levels of adherence. Adherence to medication was assessed with the eight-item Morisky medication adherence scale (MMAS-8) [38]. The MMAS-8 asks patients to respond with “yes” or “no” to a set of 7 questions and to one 5-point Likert scale question. The score for full adherence is 8, with lower scores indicating a poorer level of adherence. This scale is well validated in studies of African-American populations [39]–[41].

In the Netherlands most patients with HTN are treated in primary care practices. In those practices GPs are responsible for the management and pharmaceutical treatment of HTN. In the Dutch health care system patients can only be registered in one primary care practice, and they can only receive prescriptions for medications through a GP from this practice. In the EMRs used in this study, all prescriptions that take place under the responsibility of a GP are registered. Patients generally receive prescriptions for antihypertensive medications for a period of three months, after which they have to contact their GP for renewal of their prescription. To crosscheck MMAS-8 data on self-reported medication adherence we collected registration data from the EMR's, on the number of requests for prescription renewals patients were expected to order and the number of prescription renewals they actually ordered, for two periods: (i) the 12 months prior to their inclusion in the trial, and (ii) the 12 months after their inclusion in the trial. For each period a patient was defined as adherent if the number of prescription renewals s/he was expected to order, matched the number of requests s/he actually ordered.

Exploratory outcomes were the mean between-group differences in sodium intake and in BMI reduction at 6 months. Sodium intake was calculated from urine analysis of an overnight sample [42]. BMI was calculated from the patient's height and weight. Height and weight were measured in the absence of shoes and heavy clothing, using a validated tape rule and weighing scale respectively. Measurements were recorded to the nearest 0.1 cm and 0.1 kg.

In order to adequately characterise the study sample, we collected self-report data on socio-demographic variables (age, gender, self-identified ethnicity, educational level, employment status, religion and health insurance status), duration of HTN and some other factors.

Two trained RA's, who were blinded to the study condition, performed the baseline and follow-up assessments. All data were entered into SPSS Data Entry 4.0 (Ref: SPSS Inc., Chicago IL, US) by one data entrant and checked by another. All data entrants were blinded to the study condition.

### Sample size calculation

As described previously [34], we aimed for a sample size of 74 participants in each arm of the trial, based on α = 0.05 and β = 0.20 and an inter-cluster correlation (ICC) = 0.03 to detect a difference of 10 (SD 15) mmHg in SBP between the study arms after 6 months. We assumed an ICC of 0.03 on the basis of previous experience and the literature [43].

### Statistical analysis

We used SAS version 9.13 and STATA version 10.1 for statistical analyses. The statistician who analysed the data was blinded to the intervention assignments. The main analysis was intention-to-treat and involved a logistic regression analysis. The model included education (three levels), age, sex, duration of HTN and baseline measurement as a priori covariates. When the model included the cluster effect, we used the SAS procedure nlmixed to estimate the model and calculate treatment and control proportions predicted from the model, with education level and clusters weighted by their relative sizes and covariates set at the means in the entire sample. For the continuous variables a linear regression analysis was performed using a treatment indicator as the variable of central interest, the cluster effect as a fixed effect nested within treatment and the same a priori covariates that were used for dichotomous variables. Effects were retained in the model regardless of their statistical significance.

### Study protocol changes

Before the study began, the registered study protocol (ISRCTN35675524) was modified on the following points: (i) SBD and DBD were explicitly included as pre-specified secondary outcome measures, because of the clinical relevance [44], and this change was added to the protocol that was approved by the medical ethics committee. (ii) The planned 12-week intervals between CAHE sessions in the original protocol were shortened to 9 weeks on average, because patient education specialists argued that 12 weeks would be too long for patients to retain what was discussed with the PN. Consequently, also the time period between the baseline (T0) and the final assessment (T1) was shortened from 8-to-6 months. (iii) In contrast to the planned protocol, in our study no fixed intermediate BP measurements were conducted between the baseline- and the final assessments, since the GPs of the participating practices commented that this would be a deviation from usual care. All modifications were approved by the scientific committee of the sponsor of the study, the Netherlands organisation for health research and development (ZonMw).

## Results

**Figure 1:**
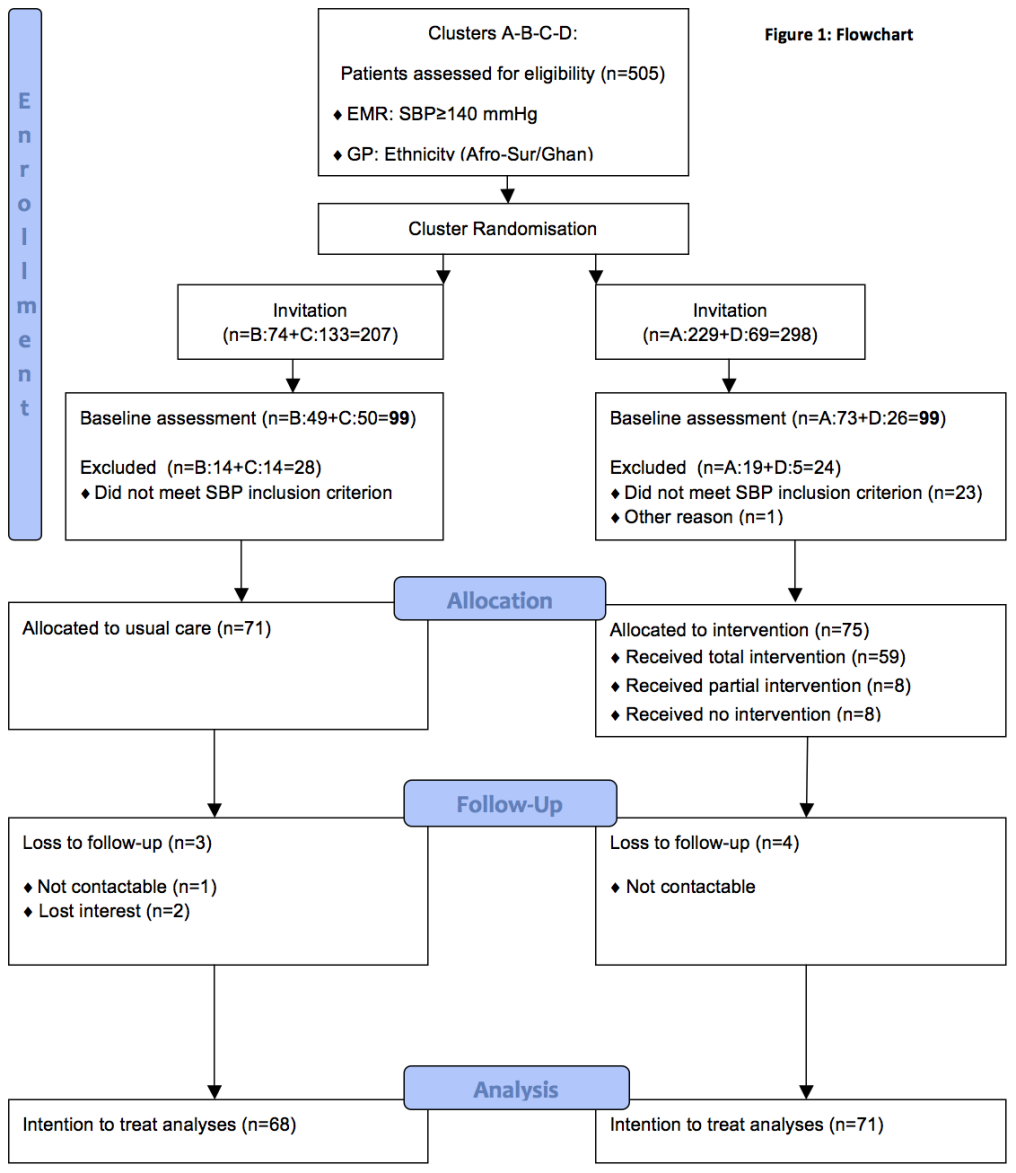
Flowchart.

Patient recruitment occurred in all four PHCCs between November 2009 and March 2010 and the intervention ran from December 2009 to October 2010 (see practice factors, Table 3). Figure 1 shows the flow of the patients through the study. In all, 505 patients who met the initial inclusion criteria were invited to participate in the study. After cross checking for inclusion criteria (SBP, self-identification, ability to speak and understand basic Dutch or English), 146 of the 198 patients who responded to the invitation were assigned to CAHE (n = 75) or to usual care (n = 71).

**Table 3:** Baseline characteristics of study practices and patients, according to treatment condition (intervention-control).

	Intervention	Control
***Practice factors at baseline***		
**Number of practices**	2	2
**Mean number of patients/practice**	10731	12017
**Mean (%) of practice population on hypertension register**	1299 (12)	1546 (13)

*Patient factors at baseline*	Intervention	Control	P value
	N = 71	N = 68	
**Mean age: years (SD)**	53.3 (10.2)	54.6 (9.5)	0.46
**Number (%) sex**			
- Female	43 (61)	30 (44)	0.052
- Male	28 (39)	38 (56)	
**Number (%) ethnicity**			
- Ghanaian	20 (28)	26 (38)	0.21
- Surinamese	51 (72)	42 (62)	
**Mean duration of stay in NL: years (SD)**	24.9 (10.6)	24.8 (9.4)	1.00
**Number (%) education level**			0.37
- Low	33 (46)	37 (54)	
- Middle	21 (30)	21 (31)	
- High	17 (24)	10 (15)	
**Number (%) employment status** a			0.24
- Paid work	48 (69)	40 (59)	
- Unpaid work	2 (3)	-	
- Unemployed/disabled	13 (19)	20 (29)	
- Retired	7 (10)	8 (12)	
**Number (%) religion**			0.58
- Christian	55 (77)	49 (72)	
- Other	4 (6)	7 (10)	
- No religion	12 (17)	12 (18)	
**Mean duration of hypertension: years (SD)**	8.9 (9.3)	9.4 (8.3)	0.79
**Systolic blood pressure: mmHg**			
- Mean (SD)	156.7 (12.3)	155.2 (10.7)	0.43
- Number (%) 140–159.9	45 (63)	47 (69)	0.76
- Number (%) 160–179.9	23 (32)	19 (28)	
- Number (%) ≥180	3 (4)	2 (3)	
**Diastolic blood pressure: mmHg**			
- Mean (SD)	91.0 (9.6)	89.6 (9.4)	0.37
- Number (%) <90	29 (41)	33 (48)	0.54
- Number (%) 90–99	30 (42)	27 (40)	
- Number (%) 100–109	11 (15)	6 (9)	
- Number (%) ≥110	1 (1)	2 (3)	
**Body Mass Index: kg/m^2^**			
- Mean (SD)	31.1 (5.3)	31.1 (4.8)	0.95
- Number (%) normal weight (<25)	4 (6)	5 (7)	0.38
- Number (%) overweight (25–30)	26 (37)	31 (46)	
- Number (%) obese: (≥30)	41 (58)	32 (47)	
**Number (%) without medication prescription** b	19 (27)	7 (10)	0.01
**Number (%) without Public Health insurance** c	4 (6)	0 (0)	0.047

a Missing values: (1).

b Self-reported.

c Due to undocumented status.

### Non-compliance with intervention and loss to follow-up

Sixteen of the 75 patients (21%) who were treated in intervention sites missed at least one of the three intended CAHE sessions. Eight of them missed all three sessions.

Data on primary and secondary outcomes were complete for all patients at baseline (n = 146), and for 95% (n = 139) of the patients at six months follow-up. Reasons for loss to follow-up were: not contactable (n = 4, intervention and n = 1, control) and no longer interested in participation (n = 2, control).

### Patient characteristics

Table 3 presents the baseline characteristics for both groups and they were similar at baseline, except for medication prescription and insurance status (p<0.05). In the intervention group, 19 patients were treated for hypertension without medication, versus 7 in the control group. Four patients in the intervention group had no health insurance because of their undocumented status. The practices had funding for the treatment of undocumented patients.

### Effect of the intervention on the between-group difference in the proportion of patients with a SBD reduction of at least 10 mmHg

After six months, the SBP level had decreased by at least 10 mmHg among 48% of the patients in the intervention group, compared to 43% of the patients in the control group. After adjustment for pre-specified covariates of age, sex, duration of HTN, education, baseline BP and clustering effect, the estimated between-group difference in the proportion of patients with a SBP reduction of at least 10 mmHg was not significant (OR: 0.42; 95% CI: 0.11 to 1.54; P = 0.19) (Table 4).

**Table 4:** Proportion of patients with systolic blood pressure reduction of ≥−10 mmHg and non-adherence to medication according to treatment condition (intervention-control) and unadjusted and adjusted odds ratios (without and with clustering effect) at six months past baseline.

	Valid Number (baseline and follow-up)		Outcome Number (%) at baseline		Outcome Number (%) at follow-up		Unadjusted Odds Ratio (intervention/control)		Adjusted Odds Ratio (intervention/control)^b^^,^ d		Adjusted Odds Ratio (intervention/control)^c^^,^ d		ICC^e^
Variable	Int	Cont	Int	Cont	Int	Cont	OR (95% CI)	P value	OR (95% CI)	P value	OR (95% CI)	P value	
SBP≥−10 mmHg	71	68	n.a	n.a	34(48)	29(43)	1.24 (0.63 to 2.41)	0.535	0.94 (0.45 to 1.98)	0.865	0.42 (0.11 to 1.54)	0.190	0.0304
Non-adherence to medication^a^	57	62	33(58)	32(52)	15(26)	33(53)	0.31 (0.15 to 0.68)	0.003	0.13 (0.04 to 0.36)	0.001	0.10 (0.01 to 0.75)	0.024	0.0909

a Data gathered through electronic medical chart review (follow up one year after baseline), only among patients who had been prescribed medication.

b Adjusted for age, sex, education, duration of hypertension, baseline measurement.

c Adjusted for age, sex, education, duration of hypertension, baseline measurement and clustering effect.

d Missing values: SBD (2); medication adherence (1).

e Effect size calculated with Bartko.

### Effect of the intervention on between-group difference in SBP and DBP reduction and adherence to lifestyle and medication recommendations

After six months, the mean SBP level in the intervention group decreased by 9.95 mmHg (95% CI: 13.33 to 6.57) from baseline (156.73 mmHg) to follow-up assessment (146.78 mmHg) and in the control group by 6.26 mmHg (95% CI: 9.50 to 3.03) from baseline (155.19 mmHg) to follow-up assessment (148.93 mmHg). The mean DBP in the intervention group decreased by 5.73 mmHg (95% CI: 7.90 to 3.56) from baseline (91.02 mmHg) to follow-up assessment (85.30 mmHg) and in the control group by 1.70 mmHg (95% CI: 3.78 to −0.39) from baseline (89.59 mmHg) to follow-up assessment (87.90 mmHg). After adjustment for pre-specified covariates of age, sex, duration of HTN, education, baseline BP and clustering effect, the estimated between-group difference in improvement was statistically significant for DBP: mean change −3.01 mmHg (95% CI: −5.73 to −0.30; p = 0.03), but not for the SBP: mean change −1.69 mmHg (95% CI: −6.01 to 2.62; p = 0.44) (Table 5).

**Table 5:** Mean scores for blood pressure and self-reported adherence to lifestyle recommendations and medication according to treatment condition (intervention-control) and unadjusted and adjusted differences (without and with clustering effect) between treatment groups at six months past baseline.

	Valid Number (baseline and follow-up)		Mean outcome (SD) at baseline		Mean outcome (SD) at follow-up		Unadjusted between-group difference (intervention-control)		Adjusted between-group Difference^c^^,^ e (intervention-control)		Adjusted between-group Difference^d^^,^ e (intervention-control)		
Variable	Int	Cont	Int	Cont	Int	Cont	Mean (95%CI)	P value	Mean (95%CI)	P value	Mean (95%CI)	P value	ICC^f^
Systolic blood pressure (mmHg)	71	68	156.73 (12.26)	155.19 (10.69)	146.78 (16.23)	148.93 (13.25)	−3.69 (−8.34 to 0.96)	0.119	−1.59 (−6.08 to 2.89)	0.488	−1.69 (−6.01 to 2.62)	0.444	−0.0008
Diastolic blood pressure (mmHg)	71	68	91.02 (9.61)	89.60 (9.36)	85.30 (10.93)	87.90 (9.53)	−4.03 (−7.01 to −1.04)	0.009	−2.98 (−5.81 to −0.16)	0.04	−3.01 (−5.73 to −0.30)	0.032	0.0093
Lifestyle adherence^a^	52	45	2.74 (0.73)	2.98 (0.70)	3.05 (0.52)	2.86 (0.63)	0.43 (0.16 to 0.71)	0.002	0.28 (0.07 to 0.51)	0.013	0.34 (0.12 to 0.55)	0.003	−0.0059
Medication adherence^b^	50	60	5.99 (1.95)	5.59 (2.17)	6.49 (1.69)	6.24 (1.82)	−0.15 (−0.84 to 0.55)	0.672	−0.13 (−0.69 to 0.43)	0.649	−0.09 (−0.65 to 0.46)	0.738	0.0420

a Self-reported lifestyle adherence score, only measured among patients who had received a lifestyle recommendation.

b Self-reported medication adherence score, only measured among patients who had received a medication prescription.

c Adjusted for age, sex, education, duration of hypertension, baseline measurement.

d Adjusted for age, sex, education, duration of hypertension, baseline measurement and clustering effect.

e Missing values: SBD/DBD (2), lifestyle adherence (1), medication adherence (1).

f Effect size calculated with semi partial Ω^2^.

After six months, the mean self-reported adherence to lifestyle recommendations (range 1–4) increased by 0.31 (95% CI: 0.12 to 0.50) from baseline (2.74) to follow-up assessment (2.98) in the intervention group and decreased by 0.13 (95% CI: −0.33 to 0.07) from baseline (3.05) to follow-up assessment (2.86) in the control group. A small increase in self-reported adherence to medication (range 0–8) occurred in both groups: 0.51 (95% CI: −0.03 to 1.04) from baseline (5.99) to follow-up assessment (6.49) in the intervention group and 0.65 (95% CI: 0.19 to 1.12) from baseline (5.59) to follow-up assessment (6.24) in the control group. After adjustment for pre-specified covariates of age, sex, duration of HTN, education, baseline measurement and clustering effect, the estimated between-group difference was statistically significant for the change of self-reported adherence to lifestyle recommendations: mean change 0.34 (95% CI: 0.12 to 0.55; p = 0.003), but not for the change in self-reported adherence to medication: mean change −0.09 (95% CI: −0.65 to 0.46; P = 0.74) (Table 5).

Registration data from the EMR's on the number of requests for antihypertensive prescription renewals doctors received from patients who were included in the study showed a decline in medication non-adherence rates in the intervention group, from 58% at baseline to 26% at follow-up, and a stable rate in the control group, of 52% at baseline and 53% at follow-up (adjusted OR: 0.10; 95 CI: 0.01 to 0.75; P = 0.024) (Table 4).

### Effect of the intervention on between-group difference in exploratory outcomes of sodium excretion and BMI

The mean sodium excretion between baseline and follow-up decreased from 65.81 mmol/L (SD 56.73) to 47.69 mmol/L (SD 38.94) in the intervention group and slightly increased from 54.93 mmol/L (SD 39.92) to 55.55 mmol/L (SD 48.75) in the control group (adjusted between-group difference −15.56: 95% CI: −39.36 to 8.23; P = 0.197). The mean BMI between baseline and follow-up decreased from 31.13 (SD 5.26) to 30.97 (SD 5.20) in the intervention group and increased from 31.07 (SD 4.80) to 31.13 (SD 4.81) in the control group (adjusted between-group difference −0.49: 95% CI: −1.08 to 0.10; P = 0.10).

### Additional analyses

The results of a per-protocol analysis that excluded the 5 patients who did not receive any of the intended CAHE sessions and the results of an analysis that controlled for two variables on which the intervention and the control group differed at base line (not treated with medication, no health insurance) did not change the main findings from the intention-to-treat analysis (data not shown).

## Discussion

In this study we evaluated the effect of a culturally adapted patient education intervention on blood pressure reduction and adherence to lifestyle recommendations and medications among 146 hypertensive patients of Surinamese and Ghanaian origin who receive care in Dutch primary care practices. We observed a systolic BP reduction of 10 mmHg or more in 48% of the patients in the intervention group and 43% in the control group, but the between-group difference was not statistically significant. Overall, the mean between-group differences (intervention and control) for systolic and diastolic BP reduction were −1.7 mmHg and −3 mmHg respectively, and statistically significant for the diastolic BP (P = 0.03). While the intervention was associated with a significant improvement of self-reported adherence to lifestyle recommendations (P = 0.003), it was not associated with a significant improvement of self-reported medication adherence as measured by the MMAS-8.

One potential explanation for the larger observed BP reduction in the intervention group is that CAHE led to an improved adherence to lifestyle recommendations, which is also supported by a positive trend of small improvements on BMI and sodium reduction in the intervention group. There is increasing evidence from studies conducted in the US demonstrating that comprehensive lifestyle modifications such as dietary changes and sodium reduction can lead to significant BP reduction in hypertensive patients with a larger reduction noted in blacks [45], [46]. The observed positive effect of CAHE on self-reported lifestyle adherence is all the more relevant because Dutch clinical HTN guidelines for GPs recommend lifestyle modification as the first treatment option for all individuals with diagnosed hypertension [35]. The expected effect of CAHE on self-reported medication adherence did not occur. This result is, however, inconsistent with our data on prescription refills that were registered by physicians in the EMRs of the patients, which suggested an improvement in medication adherence in the intervention group. This requires some reflection on the MMAS-8. We have selected this instrument, because it is a widely acclaimed validated self-report instrument [38], [47]. But MMAS-8 is not validated for the patient groups we studied. It is possible that MMAS-8 questions urged patients to give socially desirable answers, at baseline. The intervention group, however, was clearly educated that non-adherence is common among people with HTN; patients were advised not to be ashamed about non-adherence but to report any trouble with their medication honestly to the care providers to improve treatment outcomes. Therefore intervention patients may have responded more honestly to MMAS-8 at follow up. To further examine the effect of CAHE on medication adherence, the validity of MMAS-8 in ethnic minority populations should be investigated, and alternative objective measures, such as pharmacy refill data or MEMS, may be needed.

### Comparison with other studies

We compared our data with those of other behavioural intervention trials in hypertensive African Americans [48]–[51]. Cooper et al. conducted an RCT in which the effect of patient coaching by trained community health workers in underserved, mostly African-American patients with uncontrolled HTN was measured [48]. The study observed that intensive coaching led to non-significant reductions in the SBP and DBP (13.2 mmHg and 5.2 mmHg, respectively) as compared to minimal coaching (2 mmHg and 0 mmHg) at 12 months past baseline. A practice-based trial by Ogedegbe and colleagues on the effect of three motivational interviewing (MINT) sessions in uncontrolled hypertensive African-American patients showed at 12 months that MINT had led to a significant improvement in medication adherence without a statistically significant reduction in SBP (11.2 mmHg as compared to 5.2 mmHg) [49]. In a post hoc analysis of a 2×2 RCT, Bosworth reported that bi-monthly, nurse-administered tailored behavioral counselling sessions by telephone led to a significant SBP decrease of 5.7 mmHg relative to usual care at 12 months in non-white, mostly African-American hypertensive patients [50]. But at 24 months, only those patients who had received a combined intervention (counselling and home BP monitoring) had a significantly lower SBP as compared to those who received usual care (7.5 mmHg relative to usual care). The results for the DBP went in the same direction. The interventions that were evaluated in the previously mentioned US studies (MINT, patient coaching and counselling) show similarities with the standard approach to patient-centred hypertension education and counselling that is recommended by the Dutch clinical HTN guidelines for GPs [35]. CAHE combines the principles of patient-centred hypertension education with those of culturally competent care. We have found only one report of a recent trial that explicitly tested the effects of a culturally adapted intervention, culturally-appropriate storytelling, on BP changes in hypertensive African Americans [51]. The study found that patients with uncontrolled HTN who were assigned to the intervention group experienced significantly greater SBP reduction (6.4 mmHg) than the comparison group at six months, but no significantly greater DBP reduction (4.2 mmHg). Thus, in contrast to all other studied behavioural interventions, CAHE produced a significant reduction in the DBP (5.7 mmHg, relative to 1.7 mmHg in the control group). In line with the nurse-led MINT and patient coaching, CAHE produced a non-significant reduction in the SBD (9.95 mmHg, relative to 6.26 mmHg in the control group). Bi-monthly counselling sessions by telephone and culturally appropriate storytelling produced a larger SBP reduction than CAHE.

The results of CAHE are clinically relevant because many studies have shown that BP reductions translate into the prevention of cardiovascular complications [44], [52]. Particularly, it has been found that a 2 mmHg reduction in DBP is associated with a 15% reduction in risk of stroke or transient ischemic attack [53].

### Strengths and limitations

Some unique strengths of our study should be noted. First, to our knowledge, this cluster randomised trial is one of the first European studies evaluating the effect of a culturally adapted educational intervention on BP reduction and treatment adherence in ethnic minority patients. The study is unique in its explicit description of the intervention in addition to its potential replicability by health care providers and researchers in other settings [33], [34]. Second, the drop out rate of the randomised practices (0%) and the study participants (5%) was low, and comparable or better than those of other studies [51]. It should be noted that some of the work that has been done in preparation of the trial may have contributed to this: before the trial was designed we identified factors that could hamper the adoption of the intervention in routine care, such as organisational and provider-related barriers [33]. This made it possible to adapt the intervention and the study procedures to the organisational styles of the participating practices. Finally, although the study was relatively small and slightly underpowered, it has been conducted carefully, and the data are useful for meta-analyses of studies on the effectiveness of interventions aimed at improving BP control and adherence to treatment in hypertensive patients of ethnic minorities in Western societies.

Despite these strengths, the study also has several limitations. First, data were limited to ethnic minority patients of Surinamese and Ghanaian origin who receive care in four PHCCs in one geographic area in the Netherlands, thus limiting the generalisability of our findings. The intervention was designed to meet the specific needs of the study population. Even though the culturally specific content of the intervention may not be relevant to other ethnic minority groups, the protocol provides general instructions for patient education and makes it possible to adapt culturally specific contents to other populations. This study took place in practices and settings that are part of large consortia with significant resources and support including good facilities and PN quality. In addition, care providers in these practices may have had some cultural competence, as 63% of the people living in the catchment area were of non-Western origin. The observed intervention effects would possibly have been greater if the trial had taken place in practices without these circumstances. A second limitation is that, since the intervention was practice-based, it was not possible to conceal the allocation of the intervention from the health centres and care providers. This may have reduced the internal validity of the study. However, as described in the methods section, we took ample measures to minimise detection bias (e.g.; blinding RAs, validated devices, non-disclosure of intervention content). Another comment is that we observed relatively large improvements in outcomes in the usual care group, in particular for the SBP. This improvement may be explained by the fact that patients knew their BP management was being observed (Hawthorne effect). Moreover, collecting thorough data on self-reported adherence to life style recommendations and medications might have led all the patients to change their behaviour, thus reducing the between-group differences in the intervention effects. We have, however, no valid quantitative data to test this hypothesis. Furthermore, the interval between baseline and follow-up in the study was relatively short (six months) and therefore it is not possible to predict the long-term benefits of three CAHE sessions. Another limitation is that the comprehensive nature of CAHE makes it difficult to discern which aspects of the intervention were beneficial. Because we see a positive trend of small improvements in lifestyle-related physical outcomes, such as sodium intake and BMI, benefitting the intervention arm, we speculate that the BP reduction is a result of combined factors that were achieved through the intervention, which corresponds with the multi-factorial nature of the intervention. Finally, the relatively small sample size made it impossible to conduct sub-group analyses and to provide evidence of differential treatment responses by ethnic group, sex or age.

### Conclusions and implications

Nurse-led, culturally adapted patient education appears to have a beneficial effect on DBP and adherence to lifestyle recommendations for African-Surinamese and Ghanaian patients with uncontrolled HTN when compared with usual care.

Various studies suggest that hypertension care for populations of African origin living in Western countries requires improvement. Our study indicates, that CAHE can complement standard hypertension care and that patients of African origin with uncontrolled HTN can benefit from the intervention. The study provides clear directions on how CAHE can be delivered in general practice [33], [34], and may pave the way for the cultural adaptation of hypertension education in different health care settings. Larger studies are needed to confirm our observations and to identify which particular components of the intervention contribute to improved treatment outcomes. Similarly, future studies will have to study the long-term effects of CAHE and whether it is also applicable to and effective for hypertensive patients from other ethnic populations.

## Supporting Information

Checklist S1 **CONSORT Checklist.**
(DOC)

Protocol S1 **Trial Protocol.**
(PDF)
