# Supplementary material for: Culturally Adapted Hypertension Education (CAHE) to Improve Blood Pressure Control and Treatment Adherence in Patients of African Origin with Uncontrolled Hypertension: Cluster-Randomized Trial
Source: PLoS One. 2014 Mar 5;9(3):e90103. doi: 10.1371/journal.pone.0090103 (PMC3943841; doi:10.1371/journal.pone.0090103)
Supplement: Protocol S1 — Trial Protocol. (PDF) [file pone.0090103.s002.pdf]

Under Pressure II
evaluation of a culturally-sensitive education program promoting lifestyle changes and adherence among Afro-Surinamese and Ghanaian hypertensive patients: cluster randomized controlled trial

Research protocol
(September 2009)

PROTOCOL TITLE: Under Pressure II: evaluation of a culturally-sensitive education program promoting lifestyle changes and adherence among Afro-Surinamese and Ghanaian hypertensive patients: cluster randomized controlled trial

Protocol ID

Short title

Under (high) pressure II: Effect of culturally sensitive education in African Surinamese and Ghanaian hypertensive patients

Version

Sept 2009

Date

26-09-2009

Coordinating investigator / project leader

Dr.Joke Haafkens, AMC, General Practice, Meibergdreef 15, 1105 AZ, Amsterdam, 020-5667291 (f): 020-5669186 jahaafkens@amc.uva.nl

Principal investigator (s)

(Principal investigator / performer) Drs. Erik Beune, AMC, General Practice, Meibergdreef 15, 1105 AZ, Amsterdam, 020-5663983 (f): 020-5669186 ejbeune@amc.uva.nl

Sponsor

ZONMW, Program healthy life 2
PO Box 93245
2509 AE The Hague

Independent physician (s)

n.a.

Laboratory sites <if applicable>

n.a.

Pharmacy <if applicable>

n.a.

PROTOCOL SIGNATURE SHEET

Sponsor or legal representative:
<please include name and function>
For non-commercial research.
na
Head of Department:
<Include name and function> n.a.

Coordinating Investigator / Project Leader / Principal Investigator:
Signature: Dr.Joke A Haafkens
Senior researcher and project leader

Date: 26-09-2009

TABLE OF CONTENTS

1. INTRODUCTION AND RATIONALE
2. OBJECTIVES
3. STUDY DESIGN
4. STUDY POPULATION
4.1 Population (base)
4.2 Inclusion criteria Error! Bookmark not gedefinieerd
4.3 Exclusion criteria
4.4 Sample size calculation 122
5. TREATMENT OR Subjects Error! Bookmark not defined.
5.1 Investigational product / treatment Error! Bookmark not defined.
5.2 Use of co-intervention (not applicable)
6. METHODS
6.1 Study parameters / endpoints
6.1.1 Main study parameter / endpoint
6.1.2 Secondary study parameters / endpoints (if applicable)
6.1.3 Other study parameters (if applicable)
6.2 Randomisation, blinding and treatment allocation error! Bookmark not gedefinieerd
6.3 Study procedures Error! Bookmark not gedefinieerd.
6.4 Withdrawal of individual subjects
7. SAFETY REPORTING
7.1 Section 10 WMO event
7.2 Adverse and serious adverse events
7.3 Follow-up of adverse events
8. STATISTICAL ANALYSIS
8.1 Descriptive statistics
8.2 Univariate analysis
8.3 Multivariate analysis
8.4 Interim analysis (not applicable)
9. ETHICAL CONSIDERATIONS
9.1 Regulation statement
9.2 Recruitment and consent
9.3 Compensation for injury
9.4 Incentives (if applicable)
10. ADMINISTRATIVE ASPECTS AND PUBLICATION

10.1 Handling and storage of data and documents
10.2 Amendments
10.3 Annual progress report
10.4 End of study report
11. REFERENCES

LIST OF ABBREVIATIONS AND RELEVANT DEFINITIONS

ABR ABR form: (General Assessment and Registration form) is the application form that is required for submission to the accredited Ethics Committee (ABR = General Assessment and Registration)
AE:Adverse Event
AR:Adverse Reaction
CA:Competent Authority
CCMO: Central Committee on Research Involving Human Subjects
CV:Curriculum Vitae
DSMB: Data Safety Monitoring Board
EU: European Union
EudraCT:European drug regulatory affairs Clinical Trials GCP Good Clinical Practice
IB: Investigator's Brochure
IC: Informed Consent
IMP: Investigational Medicinal Product
IMPD: Investigational Medicinal Product Dossier
METC: Medical research ethics committee (MREC) in English: medical research ethics committee (METC)
(S) AE: Serious Adverse Event
SPC: Summary of Product Characteristics (in Dutch: official product infomation IB1 text)
Sponsor: The sponsor is the party That commissions the organization or performance of the research, for example a pharmaceutical
company, academic hospital, scientific organization or investigator. A party That Provides funding for a study but does not commission it is not Regarded as the sponsor, but Referred to as a subsidising party.
SUSAR: Suspected Unexpected Serious Adverse Reaction
DPA:Personal Data Protection Act (in Dutch: Person Protection Act Stat)
WMO: Medical Research Involving Human Subjects Act (Act Medical Research Involving Human

SUMMARY

Rationale: High blood pressure (BP) is more prevalent and has more serious complications among migrants of African descent than among Dutch. In the treatment of hypertensive patients of African descent, less optimal blood pressure values are achieved than among the native Dutch hypertensive patients. Adherence to treatment is essential for achieving good blood pressure values and lowering of the cardiovascular risk profile. Adherence is generally low among hypertensive patients and in particular among patients from ethnic minority groups. Tailored patient education can promote adherence. The aim of this project is to evaluate a protocol for 'culture-sensitive education"(CAHE), which is meant to guide patients in implementing lifestyle changes and proper use of medication. The target group (of this educational intervention) consists of Afro-Surinamese and Ghanaian patients who are treated for BP in general practice. CAHE was developed through a bottom-up approach in two previous projects, namely 'Heebroedoe' and 'Under Pressure I (OHD1).In these projects, two groups participated: those for which the information is intended (Ghanaian and Surinamese BP-patients) and providers engaged in the promotion of healthy behavior among BP patients (GPs, practice nurses (POH-ers), center assistants and others).The protocol has been tested by center assistants and POH-ers in three health centers in Amsterdam South East (OHD1). This demonstrated that the protocol is well applicable, particularly by POH-ers. These professionals have extensive experience and training in providing patient education in general.

Objectives:

Overall objective: To evaluate the effect of 'culturally-sensitive hypertension Education"(CAHE) in Afro-Surinamese and Ghanaian patients who are treated in general practice for high blood pressure and have a blood pressure above target (systolic blood pressure (SBP)> 140 mmHg and / or diastolic blood pressure (DBP)> 90 mmHg).

Primary specific objective:

1. To determine if there are significant differences in the reduction in systolic blood pressure between the intervention group (patients who have received CAHE) and the control group after eight months past baseline.

Secondary specific objectives:

2. To evaluate whether, whether there are significant differences in the degree of medication adherence between the intervention group (patients who have received CAHE) and the control group at 8 months past baseline.
3. Evaluate whether there are significant differences in the adherence to lifestyle advice between the intervention group (patients who have received CAHE) and the control group, at 8 months past baseline.

Study design: Randomized controlled trial, in which data will be collected at the level of the patient, and randomization takes place at the level of clusters (4 health centres);
Also, a process evaluation will be conducted to gain insight in factors that determine the observed effects of the intervention.

Study population: Hypertensive patients from primary care practice: Afro-Surinamese or Ghanaian (according to self-identification),> 20 years old, diagnosed with hypertension (K86, K87), systolic blood pressure> 140 mmHg and / or diastolic blood pressure> 90 mmHg. Patients are excluded if they have diabetes mellitus type 1 or 2 have, participate in other CVD trials, or if the treating physician deems participation to be inappropriate (eg because of existing comorbidities)

Intervention (if applicable): Patients in the intervention group will have three education meetings according to a protocol for culture-sensitive patient education (CAHE) with a trained POH, at an interval of three months. These patients also receive the usual guidance as provided in the health centers.

Main study parameters / endpoints: Primary outcome measure is the measured systolic blood pressure at baseline minus the systolic blood pressure after 8 months (blood pressure is electronically measured twice, for sitting patients after 15 minutes of rest, the average of the two measurements is recorded.). Secondary outcomes are changes after 8 months in the degree of adherence to medication and lifestyle advice; mediating factors that may influence how patients deal with hypertension: (beliefs about hypertension and medication / lifestyle changes, motivation for medication use and lifestyle changes, perceived self-efficacy to use medication/carry out lifestyle changes and satisfaction with care.

Nature and Extent of the burden and risks associated with participation, benefit and group relatedness:
1.
Patients will get a physical examination from research assistant (measurement of blood pressure, weight, height and waist circumference) and an interview. To determine the salt excretion at the interview patients will be asked to bring a tube of morning urine at T0
2.
Patients in the control group receive care as usual.
Patients in the intervention group will receive, in addition, three educational sessions according to a protocol for culture sensitive patient education from research nurse (three-month intervals: T1-T2-T3)
3.
In addition, in all patients BP will be measured three times and patients will be asked to fill out a brief self-report questionnaire on medication/life style compliance. (three-month intervals: T1-T2-T3)
4.
Finally, at the final measurement (post test), patients will get a physical examination (blood pressure measurement, weight, height and waist circumference) and an interview from a research assistant. To determine the salt excretion they will be asked asked to bring a tube of morning urine to the final interview (Posttest)

1. INTRODUCTION

High blood pressure (BP) is a major risk factor for cardiovascular disease (CVD). In BP patients this risk can be reduced through lifestyle changes that reduce additional risk factors for CVD reduction (smoking cessation, regular exercise, healthy diet, moderate alcohol consumption and reducing obesity) and if needed through proper medication use. (1) In Western countries the prevalence of BP is higher among people of African origin than among white populations. (2) According to the SUNSET study in the Netherlands the prevalence is 2 and 4 times, higher among African-Surinamese men and women than among Dutch men and women respectively. (3) Also high prevalences of hypertension and malignant hypertension were found among Ghanaians. (4-5) The SUNSET study found that only 14% of African-Surinamese men and 49% of African-Surinamese women in treatment for BP, had a blood pressure below the target values. A significant part of those who were treated also had additional risk factors for CVD:. Overweight, obesity, smoking, and physical inactivity (6) Among patients with BP adherence to lifestyle advice and medication is moderate (7-9) There are indications that this is even lower in ethnic minority groups, among other reasons because of cultural perceptions and practices that lead to people own medication practices and difficult communication between staff and patients. (10-14) A poorly controlled blood pressure and an unhealthy lifestyle can lead to unnecessary health damage and death. This damage can be prevented with better guidance. The GP plays a central role in the diagnosis and treatment of BP. (15) The NHG Standard Cardiovascular risk management (CVRM) considers education and advice as the main instruments for guiding patients towards changes in lifestyle and medication use. (1) Various studies have shown that the effect of education is increases the better it is tailored to the perspective, the needs and abilities of the patient. (7, 16-18) At the same time "culture-sensitive" information (CAHE) seems to have a positive effect among ethnic minority groups. (19) The recommended method for patient education in the NHG standard CVRM (the schedule of the 5 A's) does not differentiate according ethnicity. (20) The primary health centers in Amsterdam Southeast, have many patients of African descent, particularly Afro-Surinamese and Ghanaians, which is consistent with the demographic characteristics of this region. Many of these centers utilize a common protocol for the treatment and education of patients with BP, which is based on the NHG standard CVRM. In an earlier study, we examined in Ghanaian, African-Surinamese and Dutch patients from three of these health centers if cultural perceptions play a role the way thet deal hypertension and, if so, whether the existing education methods must be adapted. Based on the results, (21-25) and recommendations, (26) in a follow-up project we complemented the existing CVRM protocol (and digitized) with recommendations for culture-sensitive hypertension education (26) The annexes to the protocol also contain newly developed information materials on BP management and diet for Ghanaian and Afro-Surinamese BP patients. It also contains map with existing health promotion activities aimed at these groups in the area that can be used to refer patients. By means of pilot the revised protocol has been used for 6-8 months by POHers and CAs of three health care centers. Health care providers evaluated their experiences with implementing the protocol is. This showed that it is suitable for use in practice and in particular by POH-ers.

The present study aims to take a further step by evaluating the effect of CAGE in Afro-Surinamese and Ghanaian patients with hypertension. By means of an intervention, the protocol for CAHE will be delivered by a specially trained POH. This POH will conduct three CAHE sessions with patients with an interval of three months. The intervention will tested in a study with a cluster randomized controlled design. It is hypothesized that, through cultural tailoring, the effectiveness of the education will increase,

We expect that the intervention will have a positive effect on the systolic blood pressure and and the degree compliance to medications and lifestyle advice and on some mediating factors that affect patients' management hypertension. This way, the project hopes to contribute to reducing ethnic differences in blood pressure control and health outcomes in people with BP (particularly those of African descent)

2. OBJECTIVES

The overall objective: of this study is To evaluate the effect of 'culturally-sensitive hypertension Education"(CAHE) in Afro-Surinamese and Ghanaian patients who are treated in general practice for high blood pressure and have a blood pressure above target (systolic blood pressure (SBP)> 140 mmHg and / or diastolic blood pressure (DBP)> 90 mmHg).

The specific objectives are:

Primary specific objective:
1. To determine if there are significant differences in the reduction in systolic blood pressure between the intervention group (patients who have received CAHE) and the control group after eight months past baseline.

Secondary specific objectives:
2. To evaluate whether, whether there are significant differences in the degree of medication adherence between the intervention group (patients who have received CAHE) and the control group at 8 months past baseline.
3. To evaluate whether there are significant differences in the adherence to lifestyle advice between the intervention group (patients who have received CAHE) and the control group, at 8 months past baseline.

4. Furthermore, a process evaluation will be carried out that includes the following questions are discussed:
- Is it feasible engage patients of the ethnic monotity groups in question in a culture-sensitive education program? And there are differences in participation and feasibility between subpopulations (eg, by ethnic origin, gender, age, education)?

3. STUDY DESIGN

The study is designed as a cluster-randomized controlled trial (RCT), which will compare patients who receive CAHE and with those who only receive usual care. We will collect data on the level of the patient, but randomize at the level of health care centres. The main reason for choosing a RCT with cluster randomization instead of a RCT with randomisation at patient level is that with the latter study design contamination can easily occur between the intervention and control groups.

On the basis of an analysis of numbers of patients, four health care centers (clusters) are required for this study, two for the intervention arm and two for the control arm. Important selection criteria for health care centers are that they should treat a sufficient number of patients from the target groups and that the hypertension care takes place according to the NHG standard CVRM.

The four health centers participating in the study will be randomly assigned to the intervention arm or the control arm (1 to 1 ratio).
The randomization will be performed by an employee of the Data Management Services team of the KEBB department of the AMC who is not involved in the study and blinded to the identity of the practices. In the health centers that are assigned to the intervention arm enrolled patients will get the CAHE. In the health centers that are assigned to the control arm enrolled patients will get the usual care which is provided in the health care centers. It is assumed that intercluster variance will be low. Based on the inclusion criteria and the fact that patients are treated with the same standard NHG in all four centers, we assume that differences in the mean blood pressure of hypertensive patients from the four centers will be low. Because the intra-cluster correlation coefficient is a ratio of the low between cluster variation and the within cluster variation, the intra-cluster correlation (ICC) is estimated to be <3%.

4. STUDY POPULATION

4.1 Population (base)

Practices
A total of 240 patients who are diagnosed with hypertension and meet the inclusion criteria will be recruited from the participating general practices (health care centers). It is expected that the following practices will participate in the study: two GAZO-centers: “Reigersbos” and “Klein Gooioord” and two other centers in the 'high Bijlmer' neigborhood. We choose these practices because they are located in a region where relatively many Afro-Surinamese and Ghanaians are living. The first two centers that are part of the GAZO foundation (Healthcare centres Amsterdam South East), have an estimated number of 401 Afro-Surinamese and 75 Ghanaian patients with hypertension (K86 + K87). As other health care centers centers, 75 in Amsterdam South East we are considering Kraaiennest and a center to be determined). The latter two centers are located in the "high Bijlmer ,' the neighbourhood where the with the highest number of Ghanaian and Surinamese population in Amsterdam South East. Based on data from the SUNSET study, (6) we assume that about 63% of the patients who are treated for HTN will have a BP above the target values (> = 140/90 mm Hg at two measurements)

4.2 Inclusion criteria

Patients will be included if they meet the following criteria: Afro-Surinamese or Ghanaian (according to self-identification), older than 20 years, according to the electronic patient record diagnosed with hypertension (K86, K87), a blood pressure higher than the target values (systolic blood pressure (SBP)> 140 mmHg and / or diastolic blood pressure (DBP)> 90 mmHg)

4.3 Exclusion criteria

Patients will be excluded if they have Diabetes Mellitus type 1 or 2 diabetes. The reason is that many diabetic patients with BP are following diabetes education programs and such programs may overlap with parts of the intervention we offer in this trial. Furthermore, patients are excluded from participation, if they participate in other CVD trials or if the treating physician considers the patient not suitable for participation (eg due to existing comorbidities)

4.4 Sample size calculation

Four health care centers will be needed for the study and cluster-randomization. We assume a low between-cluster variance because of the homogeneity of demographic characteristics of the patient populations and treatment approaches of the participating health care centers. Based on the inclusion criteria and the fact that patients are treated according to the same clinical guideline (NHG), we assume little variation between the average blood pressures of hypertensive patients with an inadequately controlled BP. Because the intra-cluster correlation coefficient (ICC) is a ratio of the low between-cluster variation and within-cluster variation, we assume an ICC of <3%. The primary outcome measure will be a decrease in SBP at 8 months. A decrease of 10 mmHg (standard deviation = 15) in the intervention group compared to the control group will be considered as clinically relevant. To demonstrate this difference statistically with a reliability of the 5% chance of Type 1 error and a power of 80%, an average cluster size of 30 patients per cluster (health care centre) is needed. We strive to recruit a total 120 patients: 60 patients per treatment arm.
Assuming an average dropout rate of 50%, 240 participants will have to be recruited.

5. TREATMENT of Subjects

5.1 Investigational product / treatment

INTERVENTION GROUP: culturally sensitive hypertension education
Method
The patients participating in the intervention group will get three consultations with a practice nurse (POH) of 20-30 minutes with an interval of three months. The method evaluated in this study, culturally sensitive hypertension education (CAHE), is an addition to the standard education method (the 5 A's *) which is used to assist patients in dealing with high blood pressure and which is recommended by CVD guidelines of NHG (1).

(5 A's: 1. Ask: question regularly and systematically about the current behavior; 2. Assess: determine the willingness to bring about behavioral change; 3. Advise: strongly recommend to achieve behavioral change; 4. Assist: assisting where possible through referrals, supportive treatment or medical intervention; 5. Arrange: investigate potential conditions for relapse prevention and make follow-up arrangements.)

CAHE focuses on discussing culture-specific aspects of the patients' perspective on BP and treatment. The preliminary study showed that three sessions (consultations) often enough to essential aspects of the patients' perspective on hypertension with the patient.
After the baseline assessment the POH will make appointments for the three sessions with the participant. A week before a scheduled session, the participant will receive a written reminders from the POH. The POH will record the sessions with an audio recorder, only after permission from the patient. The recordings will be used for the process evaluation.

Contents CAHE
The first session is mainly aimed at eliciting ideas, concerns and expectations on BP and treatment, using reflective listening, giving medical information about BP, matching both perspectives, and setting a target for the follow-up session. The two follow-up sessions focus on discussing patients' experienced barriers and increasing their motivation and skills for continuing to achieve their medication and life-style targets. The approach use in these session is derived from theories the medical anthropologist Kleinman (especially the distinction between professional and lay 'Explanatory Models'), (27) and recent approaches for improving adherence in hypertensive patients. (28)

Treatment goals for the hypertension patient according to NHG clinical guideline):
• Blood pressure: reaching the following target blood pressures: SBP <140 mmHg and DBP <90 mmHg with appropriate use of medication. If necessary adjustment medication in consultation with GPs to reach the targets. If target with three agents are not achieved, SBP decrease of at least 10 mmHg acceptable.
• Lifestyle Changes (if necessary): Quit smoking, a maximum of 2 alcoholic drinks per day (women) or 3 (males); regular physical exercise: min 5 days a week 30 minutes biking / brisk walking / gardening etc.; Proper diet: low fat , min 1 time per week fatty fish, 200 grams of vegetables and 2 pieces of fruit daily, little salt; Optimal weight: BMI <25 kg/m2, waist circumference 80 cm (women) / 94 cm (men). In order to achieve the lifestyle adjustments it may be necessary as an intermediate to refer the patient to a health care professional specialized in the field.

The recommended method for providing CAHE consists the method described in the NHG guideline CVRM, supplemented with specific questions from the CAHE protocol that was developed in the previous study ("Under Pressure 1)" . (26) In addition to this information leaflets can be yused which are tailored to the needs of Ghanaian and Surinamese

hypertensive patients and a map of neighbourhood facilities that support lifestyle changes for for Ghanaian and Surinamese.
The session with Afro-Surinamese patients will be held in Dutch and with Ghanaian patients in English or if they prefer this in Dutch or in a combination of Dutch and English.
After three educational sessions the patients in the intervention group will be given the regular hypertension care.

Box 1 contains a summary of the sessions.

Box 1: Overview phases culturally sensitive hypertension education

**Sessions/topics**	**Eliciting patiënt-perspectief**[3]	**Information from medical perspective** [4]	**Tuning the two perspectives**	**Increasing motivation to change behaviors**
Session 1:<br>What is hypertension?<br><br>Communication patient health care provider	Hypertension<br><br>Communication-barriers	Hypertension	Understanding of hypertension	
Session 2 and 3:<br>Managing hypertension<br><br>Education/treatment	-BP control<br>- Targets[1] BP;<br>Lifestyle changes [2]	-BP control<br>- Targets[1] BP;<br>Lifestyle changes [2]	Relate to patiënt-perspective[3]<br>+<br>Joint decision making	Motivational strategies[5]

The health care professional providing CAHE:
In two intervention centers a POH will be responsible for providing CAHE. This POH will be appointed specifically for the purposes of this study and will receive a clear protocol and training and will be instructed not to provide information about CAHE to colleagues.

CAHE sessions with patients in the intervention group should preferably be held at the health care centers where the patient is being treated. The POH who is to be recruited for this study, must be trained in the principles of patient counselling according to the 5 A scheme. The specific training the POH will receive for the purpose of this consists of information about BP in patients of African origin, a training in how to work with the CAHE protocol in patients from the target groups. Use is made of the training manual that has been developed in the previous project (under-pressure-I).
After the T0 assessments, patients in the intervention group will receive three appointments for CAHE sessions with the POH (2 weeks, 3 ½ and 6 ½ months after T0 measurement) At these intervals the POH will also measure patients' BPs with an electronic device (Omron 705-IT) according to a standardized method, the treating POH. This is a standard component of hypertension education, both in the experimental intervention protocol and in the NHG protocol. At these points, we will also measure the self-reported adherence to medications and lifestyle advice through a short questionnaire. The POH will ask the patient to complete these lists in the waiting room.

CONTROL GROUP
The POH's in control centers give patients the usual care. The patients in the control group will be given an appointment at 3 ½ and 6 ½ months after the T0 measurement to get their repeat prescriptions for anti-hypertensive medications. A blood pressure reading will be performed by the treating POH with an electronic device (Omron 705-IT) according to a

standardized method. In all patients in the control group after 3 ½ and 6 ½ months self-reported adherence to medication and lifestyle advice will be measures via a short questionnaire. The POH will ask the patient to complete these lists in the waiting room

6. METHODS

6.1 Study parameters / endpoints

6.1.1. Main study parameter / endpoint
Difference between baseline systolic blood pressure and systolic blood pressure at 8 months past baseline.

6.1.2. Secondary study parameters / endpoints (if applicable)

Difference between baseline and 8 months past baseline regarding the following measures:
- Adherence to medication
- Adherence to lifestyle advice
- Mediating factors that may affect the way in which patients manage hypertension:
o beliefs about hypertension and medication / lifestyle changes
o motivation to use / medication to carry out lifestyle changes
o self-efficacy to use / medication to implement lifestyle changes
- Satisfaction with care
- Baseline demographics of potential confouders, such as age, gender, ethnicity, environment and other socio-economic circumstances are included in the study.

6.1.3 Other study parameters (if applicable)
A process evaluation will be conducted to provide insight into factors that may be relevant to determine the effect of the intervention:
- is the study conducted as planned (fidelity)?
- participation in the study
- Information on refusals to participate
- reasons for discontinuation in participating in scheduled consultations in study arms
- information about obstacles that arise in CAHE sessions (via tape recordings)
- Explanatory models, perceived barriers and facilitators for following medication and lifestyle advice; (via some in-depth interviews with patients from intervention and control groups)

6.2 Randomisation, blinding and treatment allocation

Randomization takes place at the level of the health care centers. There are four health centers (clusters) needed for this study, two for the intervention arm and two for the control arm. Through a computer designed randomization list recruited centers will be divided into control centers and intervention centers. Once the centers have given their consent to participate, the randomization will be performed by an employee of the AMC Data Magement Services team KEBB department of the AMC who is not involved in the study and blinded to the identity of the practices

Blinding
In order to ensure blinding the measurements will be carried out as follows:
1. For the purpose of the beginning (T0) and the final measurement a research nurse will blood pressure measurements and physical examinations in all participating health car centers and collect the batch of morning urine from the patients. For blood pressure measurements validated electronic devices will be used (Omron 705-IT). A research assistant will carry out the study’s questionnaire to assess other outcome measures. The research nurse and research assistant will be blinded to the study arms to which centers are allocated..
2. In the two intervention centers a POH will be responsible for delivering CAHE. The POH will receive a clear protocol and training (see 5.1). S/he will also be instructed to provide no

information about the study or the patient education to colleagues. The POH's in control centers will give patients the usual care. The blood pressure measurement is a standard component of hypertension education, both in the intervention and usual care. The interim blood pressure measurements at 3, 6 and 9 months, will be carried out by the attending POH's with the above-described electronic electronic devices. The use of a standardized measurement methods restricts the possibility of ascertainment bias.
3. There will be blinding during while entering and analyzing the data. All data from the intervention and control groups are stored in one data set and the researchers who are cleaning the data will be blinded to the study arms.

6.3 Study procedures

DATA COLLECTION
Medical records
After patients have been included in the study, retrospective data from the patient's Electronic Medical Record (EMR) will be recorded: age, blood pressure (SBP / DBP) of the previous two consultations, prescribed medication, and if present, data concerning additional risk factors for CVD (number of minutes of exercise per week, diet, salt intake, smoking, alcohol consumption, height / weight, BMI, waist circumference, glucose and lipids (total cholesterol, HDL-cholesterol, TG, LDL), and co-morbid conditions in the patients history such as CVD (myocardial infarction, congestive heart failure, transient ischemic attack, stroke), kidney (renal failure, microalbuminuria), dementia, psyche (depression, schizophrenia), liver diseases.

Pre and post-test
Patients in the intervention group and the control group will participate in a series of pre- and a post-intervention measurements which will be conducted by a specially trained research nurse. Baseline measurements will take place as soon as possible (within two weeks) after inclusion and final measurements between four-to-six weeks after the patients' last educational session/ repeat prescription. All the measurements will be carried out in one of the four health care centers that participate in the study and, if possible, the health care center where the patient is registered.The measurement consists of: 1. A physical examination, 2. Identification of additional risk factors for CVD and 3. the administration of a questionnaire.

Ad 1. Physical examination:
Blood pressure: Systolic and diastolic blood pressure will be measured twice electronically, in patients who are seated with a 15 minute interval. The average of the two measurements will be recorded. (1)
Furthermore, weight, height and waist circumference will be measured (+ BMI calculated). The physical examination will be performed in a standardized way, in the health center where the patient is being treated. For these measurements, the protocol of the earlier SUNSET study will be used. (29)

Ad 2. Additional risk factors for CVD
Through a short questionnaire information will be collected about the current status of modifiable risk factors for CVD: number of minutes of exercise per week, diet, smoking, alcohol consumption. For this, questions from the SUNSET study will be used (29). Data on salt use are will be collected through a morning urine sample (lab-determination: Sodium and Creatinine).

Ad 3. Interview

The research nurse or interviewer will collect questionnaire data, immediately following the physical examination. The interview will take place in the participant's preferred language (English or Dutch). The interview includes questions about:
1. Adherence to treatment with regard to medication
2. Adherence regarding lifestyle advice.
3. Health: patients ideas about their own health.
4. Hypertension: ideas about hypertension and the causes, the way one deals with hypertension, motivation to do something about hypertension, confidence (self-efficacy) in managing ;hypertension.
4. Medication; ideas about medication in general, ideas about antihypertensive medications, experiences in taking antihypertensive medications, intentions (motivation) for following medication advice.
5. Lifestyle advice: ideas about lifestyle changes, acquired experience in dealing with lifestyle advice, intentions (motivation) on the compliance of lifestyle advice: This involves exercise per week, diet, salt intake, smoking and alcohol consumption.
6. Treatment: Satisfaction with treatment/care.
During the baseline assesment, the following demographics of the patient will be collected: age, gender, ethnicity (birth parents and grandparents; self-identification), education, occupation, income, marital status, duration of residence in the Netherlands, hypertension duration, health insurance. This part of the questionnaire will not be included in the post-test. During the post-test it will be assessed if stressful events have happened between account possible T0 and the final assessment. This information can be used in the final analysis of the data in order to assess potential confounders or effect modification (eg change in income, marital status).

Underneath we will provide more detailed information on the questionnaires that will be used in the interview. Where possible standardized validated questionnaires will be used:
1. To measure self-reported measure medication compliance, we will use the four-item scale Morisky. (30) To measure social support for adherence to treatment we will use the validated social support scale, from the MOS (Medical Outcome Survey) which had been used by Ogedebge (31) in a similar study.
2. To measure compliance with regard to lifestyle advice, we use a modified Morisky scale per lifestyle advice. (30)
3-5. To measure items that are mention in ad 3 to ad 5 above, we will use the High Blood Pressure Questionnaire (Utrecht University) that has been used in earlier Dutch research. (17.32) The High Blood Pressure questionnaire consists of components of validated international questionnaires: the Illness Perception Questionnaire (IPQ) which measures perceptions of disease, (33) the Beliefs about Medication Questionnaire, which measures perceptions on medication, (34,35) the Stages of Change Readiness and Treatment Eagerness Scale (SOCRATES) that measures behavioral intentions s, (36) a scale by Lorig measuring self-efficacy. (37) To the High Blood Pressure Questionnaire we will add a number of questions that are based on insights gained from our previous project Heebroedoe. (22-25) It is also possible that we choose a questionnaire which included similar elements and was previously used in the SUNSET study, conducted by the Department of Social Medicine of the AMC (29).
6. To measure satisfaction with care we consider using the Migrant QUOTE. (38) This is a validated questionnaire developed by NIVEL and the Department of Social Medicine of the AMC and which is adapted for use in migrants.

The selected questionnaires are, generally, not yet validated for the specific ethnic groups participating in this research. If we are still able find equivalent questionnaires that have been validated for non-Western populations, eg from the SUNSET study, we will prefer to select those questionnaires.

T1-T2-T3 measurement

After the physical examination for the T0 assesment, three appointment will be made with patients in the intervention for the three CAHE sessions. A first after 2 weeks and on two subsequent times (intervals of 3 months).
After each session in patients in the intervention group blood pressure measurements performed by the treating POH (T1), (T2) (T3). In patients in the control group BP measurements will be conducted (after 3 ½ and 6 ½ months) by their treating POH in a standardized manner. On those occasions self-reported adherence to medication / lifestyle advice will also be measured with the Morisky scale and adapted Morisky scale, respectively. The treating POH will ask the patient to fill in this list in the waiting room

Medication Use
Besides self-reported medication adherence, medication adherence will also be evaluated using pharmacy data. Thereby the number of times in which a prescribed recipe for anti-hypertensives is picked up in time will be used as a measure. Thi9s indicates 'refill adherence ". Refill adherence is defined as the number of days for which antihypertensives are supplied divided by the total number of days after which the patient collects his/her repeat prescription). (39) A patient is defined as dherent if t he repeat prescription is filled within 7 days of the designated date. This method is also used in other research. (40)

Process Data
For process data on participation in the study and possible reasons for discontinuation of treatment in both study arms, the EMR data will be used. About 6-10 participants in the intervention group will be asked to participate in an in-depth interview. During the interview information will be collected on issues that have been tackled during CAHE and any obstacles that arose in these sessions.
About 6-10 participants from the control group will also be asked to participate in an in-depth interview to explore that experiences of barriers and facilitators for adhering to medication and lifestyle advice.

Costs
The consultations in the context of the intervention do not differ from the normal care and can be reimbursed by the patients'health insurance. When patients are not insured, an alternative solution for reimbursement will be sought.
To explore the personnel costs the intervention entails an the POH and other practitioners will be asked to record the time they spent on measurements and patient education. Time spent by health care professionals in the intervention and control group will be compared. Through the qualitative interviews with patients we will seek to get an impression of the costs patients had in participating in the intervention: indirect costs of participation in measurement and intervention sessions such as travel expenses or costs associated with lifestyle changes.

6.4 Withdrawal of individual subjects
Participants can, as soon as they want, at any time, for any reason, withdraw from the study. This will not have any consequences for to their normal medical treatment and care. The researcher may decide, if necessary after consultation with the attending physician, to end the study participation of a patients for pressing medical reasons.

7 SAFETY REPORTING

7.1 Section 10 WMO event

In accordance to section 10, subsection 1, of the WMO, the investigator will inform the subjects and the reviewing accredited METC if anything Occurs, on the basis of Which it appears that the disadvantages of participation May Be Significantly greater than was foreseen in the research proposal. The study will be suspended pending further Top review by the accredited METC, except insofar as suspension would jeopardize the subjects' health. The investigator will take care That All subjects are kept informed.

7.2 Adverse and serious adverse events

Adverse events are defined as any undesirable experience occurring to a subject during a clinical trial, Considered Whether or not related to the investigational drug. All adverse events reported spontaneously by the subject or observed by the Investiga ¬ tor or his staff will be recorded.

A serious adverse event is any untoward medical occurrence or effect That at any dose results in death;
- Is life threatening (at the time of the event);
- Requires hospitalization or prolongation or Replace existing inpatients' hospitalization;
- Results in persistent or significant disability or incapacity;
- Is a congenital anomaly or birth defect;
- Is a new event of the trial likely to affect the safety of the subjects, zoals an unexpected outcome of an adverse reaction, lack of efficacy of an IMP used for the treatment of a life threatening disease, major safety finding from a newly completed animal study, etc.

All SAEs will be reported to the accredited METC That approved the protocol, According to the requirements of That METC.

7.3 Follow-up of adverse events

All adverse events will be followed until they have abated, or until a stable situation has Reached leg. Depending on the event, follow up May require additional tests or medical procedures As indicated, and / or referral to the general physician or a medical specialist.

8 STATISTICAL ANALYSIS
8.1Descriptive statistics 8.2 Univariate analysis Multivariate analysis 8.3 8.4 Interim analysis (if applicable)

8.1 Descriptive statistics
Quantitative data: Primary outcome measure: The main purpose of this study is to measure the effect of the intervention on systolic blood pressure in mmHg (difference between systolic blood pressure (SBP) measured at baseline and after 8 months)

Qualitative data: For a better understanding of the effects of the intervention a process evaluation was carried out, on the basis of the aforementioned qualitative data. This data will be transcribed in whole or in part. The analysis will be carried out using the computer analysis program MAXQDA, based on predefined questions and codes (framework analysis): topics that are addressed in intervention sessions; barriers that arise in thethese sessions;- factors impeding or promoting adherence to medication and lifestyle advice;- explanatory model of hypertension. Data on the number and reasons for refusals to participate in CAHE sessions or to terminate these sessions will be analyzed for frequency and content.

8.2 Univariate analysis
Analysis of the primary outcome data: the systolic blood pressure measured at baseline minus the systolic blood pressure measured at 8 months past baseline.
All data entries will be controlled at random to avoid potentially incorrect codings. Univariate analyses will be conducted on the corrected database to compare variable distributions and identify positive or negative outliers. The analysis of the primary outcome measures will be carried out according to the 'intention to treat' principle The primary outcome measure is calculated for each patient. Then the average within-group difference will be calculated for patients in the intervention group and the control group. Subsequently, the between-group difference between the calculated within-group differences will be calculated with a corresponding confidence interval. A decrease in systolic blood pressure by 10 mmHg (standard deviation = 15) in the intervention group as compared to the control group, with a confidence level of 5% probability of a Type 1 error, is considered to be clinically relevant. The analysis will use univariate and adjusted multilevel analysis.

The secondary data are analyzed in the same way.

If possible, separate subgroup analyzes will be carried out by ethnic group and gender. For all analyzes, a p value of 0.05 is used as the critical value. In subgroup analyzes will be an adjustment to the p value for the number of analyzes performed.

8.3 Multivariate analysis
Differences in the blood pressure values between treatment arms will be analyzed using a mixed model regression analysis that will take the dependence of the data and the intraclass correlation between centers into account. Corrections will also be performed for any differences in baseline characteristics of patients between the treatment arms.

8.4 Interim analysis (if applicable)
NA

9 ETHICAL CONSIDERATIONS

9.1 Regulation Statement

This study will be conducted according to the principles of the Declaration of Helsinki (October 2000) "and according to the (Dutch) Medical Research Involving Human Subjects Act (WMO) '

9.2 Recruitment and consent

Recruitment

As a first step in the selection process potentially eligible patients will be selected on the basis of a list of data from the electronic medical records (EMR)from the participating health centers. The selection of patients will be based on the previously described inclusion and exclusion criteria.

The EMRs contain no information on ethnicity, but this is an essential variable of this study. Therefore, as a second step in the selection, based on the selection procedure in step 1, a list of names of names will be produced without further information about the patients. From this list people with Afro-Surinamese and Ghanaian names will be selected. This method was previously used in the project Heebroedoe and projects at the Department of Social Medicine of the AMC. Therfore, a list of common Afro-Surinamese and Ghanaian names, is already available. In the latter studies the selection of patients by name was made by a GP or practice nurse. In the present study, it is of great importance to avoid selection bias. Therefore an independent person, who does not know the patients, will be ask to select patients with Ghanian and Afro-Surinamese names from the list name matching. Only in case of doubt a GP or the POH may be consulted about the ethnicity of the patient. Once the list is ready the attending physician will be asked, if the selected patients are suitable to participate in the study, based on their health. During selection procedure additional information (retrospective, if available) will be recorded from the EMRs of potentially eligeable patients on additional risk factors for CVD (f minutes of exercise per week, diet, salt intake, smoking, alcohol consumption, height / weight, BMI, waist circumference, glucose and lipids (total cholesterol , HDL-cholesterol, TG, LDL), and other conditions such as myocardial infarction, congestive heart failure) TIA, stroke, renal failure, microalbuminuria), dementia depression, schizophrenia or liver disease). This additional information will only be used, however, after the patient has given informed consent to participate in the study. The selected eligible patients will receive an invitation letter to participate in the study in English (Ghanaians) or Dutch (Suriname) on behalf of their GP during their next appointment or by mail. In this letter patients will be informed about the study and invited to participate. The invitation letter contains a form with a free return envelope in which the patient can indicate if he / she wishes not to be contact about the study. The patients who have not responded within two weeks will be contacted by telephone by the investigator who will provide them with information about the study and asks them if they are willing to discuss potential participation in the study.

All written information about the study is available in the Dutch language (see Annex) and English.

Informed Consent

The patients in the intervention group and the control group will receive, prior to the T0 measurement, an oral explanation of the invitation letter with the opportunity to ask questions about the purpose and the further course of the study and the measurements. The participant is then asked if s/he want to sign the. "Informed consent" form. This informed consent is used to ask for permission:

- Participation in the study at T0 (baseline measurement)
- Participation in the follow-up contacts
- Permission to collect data relating from the EMR related to the treatment of hypertension.

The informed consent form is available in the Dutch language (see Annex) and English

9.4 Compensation for injury
Because there are no health risks connected to the participation in this study the Medical Ethics Committee of the AMC has granted an exemption from the requirement to buy a special insurance which covers participants for any risks associated with participation research.

9.5 Incentives (if applicable)
After the last assessment, all participants will receive a small incentive reward for participating in the study in the form of a present.

10 ADMINISTRA TIVE ASPECTS AND PUBLICATION

10.1 Handling and storage of data and documents

The participants in the study will be assigned a research code, so that the data are anonymous and cannot be linked to individual participants. The key to the research code, will be kept by the principal investigator. There will be strict blinding to the identity of the research participants and conditions during data management and analysis. All data from the intervention and control groups are stored in one dataset and data cleaning and management of will take place completely blinded.

10.2 Amendments

Amendments are changes made to the research after a Favourable opinion by the accredited METC leg has given. All amendments will be notified to the METC That gift a favored-opinion. Sections marked in Yellow indicate an amendment to collect urine samples from the patients which was granted by the METC.

10.3 Annual progress report

The sponsor / investigator will submit a summary of the progress of the trial to the accredited METC once a year. Information will be provided on the date of inclusion of the first subject, numbers of subjects included and numbers of subjects that have completed the trial, serious adverse events / serious adverse reactions, other problems, and amendments.

10.4 End of study report

The investigator will notify the accredited METC of the end of the study within a period of eight weeks. The end of the study is defined as the last patient's last visit.

In case the study is ended prematurely, the investigator will notify the accredited METC.

Within one year after the end of the study, the investigator / sponsor will submit a final study report with the results of the study, such as any publications / abstracts of the study, to the accredited METC.

11. REFERENCES

1. NHG-Standaard Cardiovasculair risicomanagement:
http://nhg.artsennet.nl/uri/?uri=AMGATE_6059_104_TICH_R183129611676033

2.Cappuccio FP. Ethnicity and cardiovascular risk. Variations in people of African ancestry and South Asian origin. J Hum Hypertens 1997; 11(9):571-576.

3.Agyemang, C; Bindraban, N; Mairuhu, C; Montfrans, G; Koopmans, R; Stronks, K. Prevalence, awareness, treatment, and control of hypertension among Black Surinamese, South Asian Surinamese and White Dutch in Amsterdam, the Netherlands: the SUNSET study. Journal of Hypertension, 2005;23(11):1971-1977

4.van den Born BJ, Koopmans RP, Groeneveld JO, van Montfrans GA. Ethnic disparities in the incidence, presentation and complications of malignant hypertension. J Hypertens 2006; 24(11):2299-2304.

5.Agyemang,C.; Bruijnzeels,M.A.; Owusu-Dabo,E Factors associated with hypertension awareness, treatment, and control in Ghana, West Africa. Journal of Human Hypertension 2005; J Hum Hypertens 20(1): 67-71

6. Agyemang C, van Valkengoed I, Koopmans R, Stronks K. Factors associated with hypertension awareness, treatment and control among ethnic groups in Amsterdam, the Netherlands: the SUNSET study. J Hum Hypertens. 2006 Nov;20(11):874-81. Epub 2006 Aug 24.

7. Chobanian AV, Bakris GL, Black HR, Cushman WC, Green LA, Izzo JL Jr, Jones DW, Materson BJ, Oparil S, Wright JT Jr, Roccella EJ; The Seventh Report of the Joint National Committee on Prevention, Detection, Evaluation, and Treatment of High Blood Pressure: the JNC 7 report. JAMA. 2003 May 21;289(19):2560-72

8.Sabaté E. Adherence to long-term therapies: evidence for action. Geneva, Switzerland: World Health Organization; 2003. http://www.who.int/chronic_conditions/adherencereport/en/index.html

9. Fahey T, Schroeder K, Ebrahim S. Interventions used to improve control of blood pressure in patients with hypertension. *The Cochrane Database Syst Rev* 2003

10. Heurtin-Roberts S, Reisin E. The relation of culturally influenced lay models of hypertension to compliance with treatment. Am J Hypertens. 1992 Nov;5(11):787-92.

11. Bosworth HB, Dudley T, Olsen MK, Voils CI, Powers B, Goldstein MK, et al. Racial Differences in Blood Pressure Control: Potential Explanatory Factors. The American Journal of Medicine 2006; 119(1):70.

12.Charles H, Good CB, Hanusa BH, Chang CC, Whittle J. Racial differences in adherence to cardiac medications. J Natl Med Assoc 2003; 95(1):17-27.

13. Morgan M. The significance of ethnicity for health promotion: patients' use of anti-hypertensive drugs in inner London. Int J Epidemiol 1995; 24 Suppl 1:S79-S84.

14. Brown CM, Segal R. Ethnic differences in temporal orientation and its implications for hypertension management. J Health Soc Behav 1996; 37(4):350-361.

15. Burgers JS, Simoons ML, Hoes AW, Stehouwer CD, Stalman WA. Guideline 'Cardiovascular Risk Management'; Ned Tijdschr Geneeskd. 2007 May 12;151(19):1068-74.

16. Boulware LE, Daumit GL, Frick KD, Minkovitz CS, Lawrence RS, Powe NR. An evidence-based review of patient-centered behavioral interventions for hypertension. American Journal of Preventive Medicine 2001; 21(3):221-232.

17. Theunissen NC, de Ridder DT, Bensing JM, Rutten GE. Manipulation of patient-provider interaction: discussing illness representations or action plans concerning adherence. Patient Educ Couns. 2003; 51(3):247-58

18. van den Borne HW. The patient from receiver of information to informed decision-maker. Patient Educ Couns. 1998; 34**:** 89–102

19. Middelkoop BJ, Geelhoed-Duijvestijn PH, van der Wal G. Effectiveness of culture-specific diabetes care for Surinam South Asian patients in the Hague: a randomized controlled trial/controlled before-and-after study. Diabetes Care. 2001 Nov;24(11):1997-8

20. De Backer G, Ambrosioni E, Borch-Johnsen K, Brotons C, Cifkova R, Dallongeville J, Ebrahim S, Faergeman O, Graham I, Mancia G, Cats VM, Orth-Gomér K, Perk J, Pyörälä K, Rodicio JL, Sans S, Sansoy V, Sechtem U, Silber S, Thomsen T, Wood D; European guidelines on cardiovascular disease prevention in clinical practice: third joint task force of European and other societies on cardiovascular disease prevention in clinical practice (constituted by representatives of eight societies and by invited experts). Eur J Cardiovasc Prev Rehabil. 2003 Aug;10(4):S1-S10.

21. E.Beune, J.Haafkens, L.Meeuwesen, Hee Broedoe (hoog bloed): opvattingen over hypertensie van Creools Surinaamse patienten in de Huisartsen Praktijk. Huisarts en Wetenschap, (2004); 47 (13):620-624.

22. Beune EJAJ, Haafkens JA, Schuster JS, Bindels PJE. `Under pressure': how Ghanaian, African-Surinamese and Dutch patients explain hypertension. J Hum Hypertens 2006; 20(12):946-955.

23. Beune EJAJ, Haafkens JA, Agyemang C, Schuster JS, Willems D.L. "How Ghanaian, African-Surinamese and Dutch patients perceive and manage antihypertensive drug treatment : a qualitatiave study". Journal of Hypertension. 2008; 26(4): 648-656

24. Beune EJAJ, Haafkens JA, Agyemang C., Bindels PJE. Perceived enablers and inhibitors of physical activity in multi-ethnic hypertensive patients: qualitative study (submitted for publication)

25. Beune E.J.A.J, Haafkens J.A. Perspectieven op hypertensie van Ghanese, Afro-Surinaamse en Nederlandse patiënten; en de wijze waarop hiermee rekening wordt gehouden in de 1e lijns gezondheidszorg: belangrijkste bevindingen en aanbevelingen. Eindverslag ZonMw-project Heebroedoe. Amsterdam; AMC; Afdeling Huisartsgeneeskunde 11-04-2006

26. "Onder hoge druk": het bevorderen van aandacht voor cultuurspecifieke hypertensievoorlichting in de eerstelijns gezondheidszorg: ZonMw projectenpoort, projectnummer 48000002; 01-05-2006 http://zonmw.collexis.net/projectsummary.asp?Kennummer=3003

27. Kleinman A, Eisenberg L, Good B. Culture, illness, and care: clinical lessons from anthropologic and cross-cultural research. *Ann Intern Med* 1978; 88(2):251-258)

28. Betancourt JR, Carrillo JE, Green AR. Hypertension in multicultural and minority populations: linking communication to compliance. *Curr Hypertens Rep.* 1999;1(6):482–488

29. Bindraban, N.R. The Cardiovascular Risk Profile of Hindustani and Creole Surinamese in the Netherlands compared to white Dutch people. Thesis. AMC, University of Amsterdam, 2006.

30. Morisky DE, Green LW, Levine DM. Concurrent and predictive validity of a self-reported measure of medication adherence. Med Care 1986;24:67-74.

31. Ogedebge G et all. An RCT pg the effect of motivational interviewing on medication adherence in hypertensive African Americans: Rationale and design. 2006; Contemporary Clinical Trials.

32. Ridder Dd, Theunissen N. De rol van ziektepercepties in therapietrouw bij hypertensie. Gedrag&Gezondheid 2003; 31(4):237-249.

33. Moss Morris R. et al. The revised illness perception questionnaire (IPQ-R). Psychol Health 2002:17:1-16

34. Horne R. Representations of medication in treatment;advances in theory and measurement. In Petrie KJ et al. editors. Perceptions of health and illness: current research and applications. Singapore: Harwood Academic Publishers; 1997: 155-88.

35. Horne R. et all. The satisfaction with information about medicines scale (SIMS); a new measurement tool for audit and research. Quality of Health Care 2001;10:135-140.

36. Trigwell p. et al. Motivation an glycemic control in diabetes mellitus. J Psychosom Res 1997; 43:307-15.

37. Lorig K. et al. Outcome measures for health education and other health care interventions. Thousand Oaks, CA, USA: Sage Publications; 1996

38. El Fakiri FS, H.J. Weide, M.G. Kwaliteit van huisartsenzorg vanuit migrantenperspectief: ontwikkeling van een meetinstrument. Utrecht: NIVEL, 2000.

39. GEC Wetzels, PJ Nelemans, JSAG Schouten et al. Electronic Monitoring of Adherence as a Tool to Improve Blood Pressure Control. American Journal of Hypertension, 2007; 20: 119 - 125

40. Willey C, Redding C, Stafford J et al. Stages of change for adherence with medication regimens for chronic disease: development and validation of a measure. Clin Ther 2000;22: 858-71

ANNEXES:

Invitation letter and informed consent form (A: intervention condition / B: control condition)

A

Amsterdam, dd – mm – yyyy

Invitation: 'health education on high blood pressure for people of African origin'

Dear

High blood pressure is a major problem in the Netherlands, especially among the African populations. People of African origin are particular at high risk with the associated conditions such as stroke.

Therefore, a new health educational programme about living with high blood pressure has been made for the African community. The education programme has been made together with researchers from the AMC, General Practitioners and with people from the Ghanaian and Surinamese communities. It consists of three health educational sessions with a practice nurse. Researchers expect that patients with high blood pressure in the Ghanaian and Surinamese communities will benefit from this new approach. Patients will learn more about what high blood pressure is and about how they can better manage their blood pressure and the medication they receive for it. To evaluate whether this new educational programme is effective, our practice will cooperate in a study from the AMC department of General Practice.

In our practice you are registered as someone with an elevated blood pressure. Therefore I kindly ask you to participate in this study.

What does participation mean to you?

If you're willing to participate, you will be asked to:

- Take part in measuring your blood pressure and weight by a researcher and in an interview in two occasions. The first appointment for this will follow shortly after you decide to take part. The second appointment will be eight months later. You will also be asked to bring a urine sample at this appointment.
- In between you will receive three coaching sessions from a specially trained practice nurse about how to manage your high blood pressure.

The measurements, the interview and the coaching sessions will take place at our practice. The regular care you already receive for your high blood pressure, apart form this study, will continue as usual.

To thank you for taking part you will receive a gift voucher of 40 euro after the completion of the study.

Why should you participate?

By participating in this study you will learn more about what high blood pressure is, how it can affect you and how you can better manage it. This may also allow you to recognize problems and questions, so that you can present them to your General Practitioner or Practice Nurse.

How can you participate?

- fill in the enclosed answering card and send it <u>within two weeks</u> (postage free envelope)
- OR: you can directly call or e-mail the research team:
  Tel: 020-5663983 or 06 20659676. E-mail: e.j.beune@amc.uva.nl

If you would first like to know more about this study you can also contact the research team. In case you do not respond to this letter the research team will contact you. They will ask if you received this letter and can provide more information about the study. You will also be asked if you like to take part.

The study has no risks. You are free to choose whether you want to participate in this study. Your decision will not have any influence on your treatment. We also want to point out to you that all the information will be kept confidential by the researcher and will be processed anonymously. You can find more information about the research in the attached information leaflet.

From this research we can learn how General Practitioners can inform their Ghanaian and Surinamese patients about high blood pressure. We will then be more able to give our services according to the wish of the patient. So I hope for your cooperation.

With kind regards, also on behalf of the researcher Drs. Erik Beune.

Dr (name + signature GP)

THE ANSWERING CARD: *Please fill in fill in the answering card and send it <u>within two weeks</u> with the enclosed postage **free envelope**. You will be called, so please give us the your telephone numbers on which you can be easily reached.*

A

Informed consent form: consent form to participate in the study on "an educational programme on living with high blood pressure for people of African origin"

Aim of the study
The aim of the study "an educational programme on living with high blood pressure for people of African origin" is to evaluate how Ghanaian and Afro-Surinamese high blood pressure patients can best be informed.

This study is important because it can improve the health of patients. This study examines whether GPs should tailor their health education specifically to hypertensive patients with a Ghanaian or Afro-Surinamese origin.

What do we ask you?
We kindly ask you to participate in this study on high blood pressure education.
If you decide to take part, in a separate appointment a researcher will measure your blood pressure and weight and will conduct an interview. You will also be asked to bring a urine sample for determining your renal function.
After 8 months this will be repeated. All activities take place the practice of the GP.

In between you will receive three comprehensive health educational sessions about high blood pressure from a specially trained nurse practitioner.
If you decide to take part in the study we will also use some data that is related to you high blood pressure (eg blood pressure medication) from your electronic medical record of the GP.

Privacy
The results of this study will be treated confidentially and anonymously.
This means that nobody knows about the answers of the questionnaires or other medical information.
Besides, this information will only be used for this study on high blood pressure and that the information will never be given to others.

Consent Statement
I, the undersigned, will volunteer to participate in the study "an educational programme on living with high blood pressure for people of African origin".
In 8 months I will be asked again to participate in a final measurement.
I have received both written and oral information about the study.
At any time of the study I can ask for additional information and stop my cooperation, without telling why.
The researchers have promised me that my personal information will be kept confidential.

Place: ___________________________ Date: _________________
Name participant: ____
Signature: ____

I, the undersigned, have provided both written and oral information about the research and promise that all information given by the participant will be treated confidentially.

Place: ___________________________ Date: _________________
Name researcher: ____
Signature: ____

B

Amsterdam, dd – mm – yyyy

Invitation: 'health education on high blood pressure for people of African origin'

Dear

High blood pressure is a major problem in the Netherlands, especially among the African populations. People of African origin are particular at high risk with the associated conditions such as stroke.

Therefore, a new health educational programme about living with high blood pressure has been made for the African community. The education programme has been made together with researchers from the AMC, General Practitioners and with people from the Ghanaian and Surinamese communities. Researchers expect that patients with high blood pressure in the Ghanaian and Surinamese communities will benefit from a new approach that takes into account the beliefs and cultural background of patients. To evaluate whether this new educational programme is effective, our practice will cooperate in a study from the AMC department of General Practice.

In our practice you are registered as someone with an elevated blood pressure. Therefore I kindly ask you to participate in this study.

What does participation mean to you?

If you're willing to participate, you will be asked to:

- Take part in measuring your blood pressure and weight by a researcher and in an interview in two occasions. The first appointment for this will follow shortly after you decide to take part. The second appointment will be eight months later. You will also be asked to bring a urine sample at this appointment. The regular care you already receive for your high blood pressure will continue as usual.

The measurements and the interview will take place at our practice.

To thank you for taking part you will receive a gift voucher of 40 euro after the completion of the study.

Why should you participate?

By participating in this study you will help to improve the health care for high blood pressure patients from the Ghanaian and Surinamese communities. After the study you will get all the information materials about high blood pressure that belong to the programme. This may allow you to recognize problems and questions, so that you can present them to your General Practitioner.

How can you participate?

- fill in the enclosed answering card and send it within two weeks (postage free envelope)
- OR: you can directly call or e-mail the research team:
  Tel: 020-5663983 or 06 20659676. E-mail: e.j.beune@amc.uva.nl

If you would first like to know more about this study you can also contact the research team. In case you do not respond to this letter the research team will contact you. They will ask if you received this letter and can provide more information about the study. You will also be asked if you like to take part.

The study has no risks. You are free to choose whether you want to participate in this study. Your decision will not have any influence on your treatment. We also want to point out to you that all the information will be kept confidential by the researcher and will be processed anonymously. You can find more information about the research in the attached information leaflet.

From this research we can learn how General Practitioners can inform their Ghanaian and Surinamese patients about high blood pressure. We will then be more able to give our services according to the wish of the patient. So I hope for your cooperation.

With kind regards, also on behalf of the researcher Drs. Erik Beune.

Dr (name + signature GP)

THE ANSWERING CARD: *Please fill in fill in the answering card and send it within two weeks with the enclosed postage* ***free envelope****. You will be called, so please give us the your telephone numbers on which you can be easily reached.*

B

Informed consent form: consent form to participate in the study on "an educational programme on living with high blood pressure for people of African origin"

Aim of the study
The aim of the study "an educational programme on living with high blood pressure for people of African origin" is to evaluate how Ghanaian and Afro-Surinamese high blood pressure patients can best be informed.

This study is important because it can improve the health of patients. This study examines whether GPs should tailor their health education specifically to hypertensive patients with a Ghanaian or Afro-Surinamese origin.

What do we ask you?
We kindly ask you to participate in this study on high blood pressure education.
If you decide to take part, in a separate appointment a researcher will measure your blood pressure and weight and will conduct an interview. You will also be asked to bring a urine sample for determining your renal function.
After 8 months this will be repeated. All activities take place the practice of the GP.

Between the two appointments the regular care you receive for your high blood pressure will continue as usual.
If you decide to take part in the study we will also use some data that is related to you high blood pressure (eg blood pressure medication) from your electronic medical record of the GP.

Privacy
The results of this study will be treated confidentially and anonymously.
This means that nobody knows about the answers of the questionnaires or other medical information.
Besides, this information will only be used for this study on high blood pressure and that the information will never be given to others.

Consent Statement
I, the undersigned, will volunteer to participate in the study "an educational programme on living with high blood pressure for people of African origin".
In 8 months I will be asked again to participate in a final measurement.
I have received both written and oral information about the study.
At any time of the study I can ask for additional information and stop my cooperation, without telling why.
The researchers have promised me that my personal information will be kept confidential.

Place: ___________________________ Date: _________________
Name participant: ____
Signature: ____

I, the undersigned, have provided both written and oral information about the research and promise that all information given by the participant will be treated confidentially.

Place: ___________________________ Date: _________________
Name researcher: ____
Signature: ____
